# Species pool size and rainfall account for the relationship between biodiversity and biomass production in natural forests of China

**DOI:** 10.1002/ece3.8838

**Published:** 2022-04-21

**Authors:** Jia‐Jia Liu, Kevin S. Burgess, Xue‐Jun Ge

**Affiliations:** ^1^ Key Laboratory of Plant Resources Conservation and Sustainable Utilization, South China Botanical Garden Chinese Academy of Sciences Guangzhou China; ^2^ Department of Biology Columbus State University, University System of Georgia Columbus Georgia USA; ^3^ Center of Conservation Biology Core Botanical Gardens Chinese Academy of Sciences Guangzhou China

**Keywords:** Ailao Mountain, Changbai Mountain, community phylogenetic dissimilarity, environmental stress, species richness, temperature, temporal scale, Xishuangbanna rainforest

## Abstract

The strength of biodiversity–biomass production relationships increases with increasing environmental stress and time. However, we know little about the effects of abiotic (e.g., climate) and biotic (e.g., species pool and community composition) factors on this trend. Whether variation in biomass production is best explained by phylogenetic diversity metrics or traditional measures of species richness also remains elusive. We compiled estimates of community composition and biomass production for tree species in 111 permanent quadrats spanning three natural forests (tropical, subtropical, and temperate) in China. Based on ~10 years of data, we compared temperature, rainfall, species pool size, and community composition in each forest each year. We estimated species richness and phylogenetic diversity in each quadrat each year; the latter metric was based on the sum of branch lengths of a phylogeny that connects species in each quadrat each year. Using generalized linear mixed‐effect models, we found that top‐ranked models included the interaction between forest and biodiversity and the interaction between forest and year for both biodiversity metrics. Variation in biomass production was best explained by phylogenetic diversity; biomass production generally increased with phylogenetic diversity, and the relationship was stronger in subtropical and temperate forests. Increasing species pool size, temperature, and rainfall and decreasing inter‐quadrat dissimilarity range shifted the relationship between biomass production and phylogenetic diversity from positive to neutral. When considered alone, species pool size had the strongest influence on biomass production, while species pool size, rainfall, and their interaction with phylogenetic diversity constituted the top‐ranked model. Our study highlights the importance of species pool size and rainfall on the relationship between phylogenetic diversity and biomass production in natural forest ecosystems.

## Graphical abstract

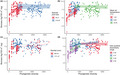

## 1 INTRODUCTION

Biomass production is a central ecosystem function (Hooper et al., 2005; Tilman et al., 2014); however, its relationship with biodiversity remains hotly debated, especially in natural ecosystems (Hagan et al., 2021). Although artificial communities typically show a positive relationship between biodiversity and biomass production (Hector et al., 1999; Huang et al., 2018; Liu, Zhang, et al., 2015; Tilman et al., 2001), the relationships found in natural communities are conflicting, including positive (Flombaum & Sala, 2008), neutral (Assaf et al., 2011), or even negative (Rose & Leuschner, 2012; Thompson et al., 2005). The sources of variation in natural ecosystems may be due to environmental effects (e.g., benign vs. harsh environments; Fei et al., 2018; Mensens et al., 2015; Pires et al., 2018) and spatiotemporal scale (Barry et al., 2019; Gonzalez et al., 2020; Li et al., 2019; Luo et al., 2019; Thompson et al., 2018). For example, the relationship between biodiversity and biomass production is neutral or even negative in benign environments, probably due to intense competition (Li et al., 2010; Xiao & Chen, 2019), whereas positive relationships in harsh environments may be due to species complementarity or facilitation (Cardinale et al., 2002; Mulder et al., 2001; Paquette & Messier, 2011; Wright et al., 2017).

At the same time, it has been long recognized that the biodiversity–biomass production relationship may also depend on the spatiotemporal scale (Chase & Leibold, 2002; Chisholm et al., 2013; Costanza et al., 2007; Fan et al., 2020; Gonzalez et al., 2020; Li et al., 2019; Luo et al., 2019; Zavaleta et al., 2010). For example, an increasing spatial scale can weaken the relationship since positive interactions between species operate at small scales (Gonzalez et al., 2020), whereas increasing the temporal scale can strengthen the relationship due in part to increased species complementarity (Cardinale et al., 2007). While there is growing evidence for the effect of spatial scale on biodiversity–biomass production relationships (e.g., Chisholm et al., 2013; Fan et al., 2020; Li et al., 2019; Luo et al., 2019; Thompson et al., 2018), temporal effects are less supported, especially in different environmental contexts (Cardinale et al., 2004, 2007; Meyer et al., 2016; Thakur et al., 2021).

Several abiotic and biotic factors may also be driving biodiversity–biomass production relationships in natural ecosystems (Hagan et al., 2021; Liu et al., 2021). Several studies indicate that climate can regulate the relationship (Ammer, 2019; Fei et al., 2018; Hisano & Chen, 2020; Jactel et al., 2018; Wang & Ali, 2021; Wu et al., 2014), which may be stronger in drier climates (Fei et al., 2018). Alternatively, biotic factors, such as species pool size and community composition, might play an essential role in the strength of biodiversity–biomass production relationships (Armitage, 2016; Burley et al., 2016; Hagan et al., 2021). For example, better hydrothermal conditions are often associated with a larger species pool, resulting in a more heterogeneous community composition (Cao et al., 2021), which can have interactive effects on the biodiversity–biomass production relationships. However, more studies are needed to disentangle these sources of variation.

In addition, the type of biodiversity metrics used can also influence our understanding of the biodiversity–biomass production relationships in natural forests. One reason might be that biodiversity is often measured by the number of species in a community (i.e., species richness; Gonzalez et al., 2020; Hagan et al., 2021; Hector et al., 1999; Tilman et al., 2001; Tilman et al., 2014), which can underestimate the variation in community composition, resulting in its relationship with biomass production being insensitive to changes in the environment and spatiotemporal scale (Hector et al., 2012). For example, species richness might remain the same even when there is a substantial change in community composition (Nabe‐Nielsen et al., 2017). In contrast, phylogenetic diversity, which is based on phylogenetic relationships among species in a community (Faith, 1992; Webb et al., 2002), may be a better indicator of the change in community composition (Donoghue, 2008). Previous studies have shown that when compared to species richness, phylogenetic diversity better explains variation in biomass production (Cadotte et al., 2009; Flynn et al., 2011; Liu et al., 2018; Liu, Zhang, et al., 2015), although estimates are often limited to homogeneous environments and on a single spatiotemporal scale (Satdichanh et al., 2019).

Variation in biodiversity–biomass production relationship and its underlying causes have been extensively studied in herbaceous communities (Craven et al., 2016; Fornara & Tilman, 2009; Grace et al., 2007; Li et al., 2019; Liu et al., 2021; Ma et al., 2010; Rose & Leuschner, 2012; Wu et al., 2014; Zuo et al., 2012). Woody communities have received much less attention, although related studies are increasing in recent years (Ali et al., 2019a, 2019b, 2020; Ali & Yan, 2017; Hanif et al., 2019; Hao et al., 2018; Jactel et al., 2018; Liang et al., 2016; Ratcliffe et al., 2017; Satdichanh et al., 2019). This study evaluates the strengths of the biodiversity–biomass production relationship for tree species in three natural mountain forests over 10 years, using both species richness and phylogenetic diversity. Our study sites represent the main climate zones of China (tropical, subtropical, and temperate zones), where we evaluated the relative importance of temperature, rainfall, species pool size, and community dissimilarity range on biodiversity–biomass production relationships. Specifically, we aimed to test the following hypotheses: (1) the effect of biodiversity will be strongest in the temperate forest (Ding & Zang, 2021), and will strengthen with time (Cardinale et al., 2007); (2) temperature, rainfall, species pool size, and community dissimilarity range will regulate biodiversity–biomass production relationships (Hagan et al., 2021; Jactel et al., 2018); (3) the phylogenetic diversity–biomass production relationship will be more sensitive to changes in environment and time than the species richness–biomass production relationship (Satdichanh et al., 2019).

## 2 METHODS

### 2.1 Data collection

**FIGURE 1:**
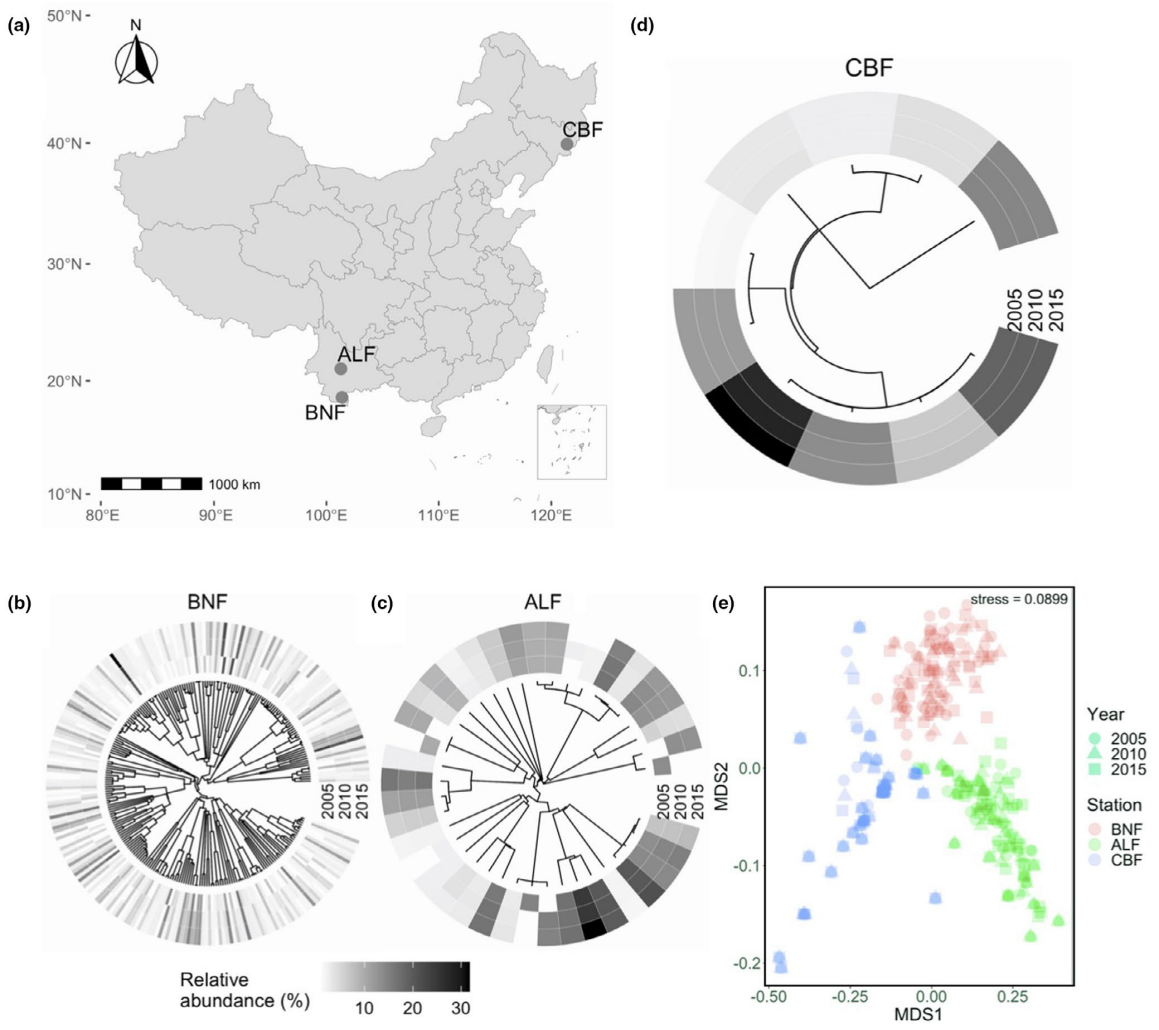
Three mature natural forests in China (a), phylogeny and mean relative species abundances of tree species in each forest (b–d), and non‐metric multidimensional scaling (NMDS) plot of the tree species communities in the three forests (e). The forests include Xishuangbanna tropical seasonal rainforest (BNF), Ailao Mountain subtropical evergreen broad‐leaved forest (ALF), and Changbai Mountain temperate deciduous coniferous and broad‐leaved mixed forest (CBF). The color gradient of the bars around each phylogeny (from grey to black) represents the species relative abundance from low to high, respectively. Each point on the NMDS plot represents a permanent quadrat's tree community with different shapes indicating different years and different colors indicating different forests. The plot was derived using a pairwise phylogenetic dissimilarity matrix generated by an abundance‐weighted phylogenetic dissimilarity metric, which weights each branch length by the abundance differences of the branch along the phylogeny of the communities. The text shows the stress value, which measures the overall goodness of fit (a stress value <0.1 indicates a strong fit in reduced dimensions)

We compiled survey data from long‐term permanent quadrats in three different mature natural forests in China (Figure 1a). The first is a tropical seasonal rainforest in Xishuangbanna (BNF; 101°20′ E, 21°95′ N). Its elevation is approximately 730 m a.s.l, the mean annual temperature is 22.7°C, and annual rainfall is 1449 mm. The soil is latosol according to the soil classification of China (Gong, 1999). Common plant species include *Pometia pinnata* and *Terminalia myriocarpa*. The second is a subtropical evergreen broad‐leaved forest on Ailao Mountain (ALF; 101°02′ E, 24°55′ N). Its elevation is approximately 2488 m a.s.l, the mean annual temperature is 12.0°C, and annual rainfall is 1804 mm. The soil type is mountain yellow–brown soil (Gong, 1999), and the common plant species include *Lithocarpus xylocarpus*, *Lithocarpus hancei*, and *Castanopsis wattii*. The third is a temperate deciduous coniferous and broad‐leaved mixed forest on Changbai Mountain (CBF; 128°09′ N, 42°40′ E). Its elevation is about 784 m a.s.l, the mean annual temperature is 3.7°C, and annual rainfall is 852 mm. The soil type is brown coniferous forest soil (Gong, 1999). The dominant plants include *Pinus koraiensis*.

For the three forests, the community composition of tree species was surveyed in permanent quadrats (10 m × 10 m) over 10 years. However, the forests varied with the number of permanent quadrats (i.e., 99 quadrats for BNF; 98 quadrats for ALF; and 37 quadrats for CBF) and the survey years (i.e., 2004–2010 & 2015 for BNF; 2005, 2010, and 2015 for ALF; and 2005, 2010, and 2015 for CBF). To address our unbalanced sampling regime, we focused on only the quadrats surveyed in 2005, 2010, and 2015 and randomly selected 37 quadrats in each forest. For each species recorded, its name was standardized or corrected according to the Flora of China (http://www.iplant.cn) and The Plant List (http://www.theplantlist.org). In total, we compiled 308 species belonging to 168 genera and 61 families. For each quadrat and each year, the biomass of each species was estimated using the allometric equation of the diameter at breast height (DBH) and/or tree height with the biomasses of different plant tissues (e.g., leaves, branches, stems, and roots; He et al., 2021). The allometric equation was either developed based on the felled standard trees in a destructive plot (FA02 table downloaded from http://www.cnern.org.cn) or obtained from Luo et al. (2015), a comprehensive database of biomass regressions for China's tree species. We summed the biomass production estimations of all species in each quadrat for each year as community biomass production (kg/100 m^2^). Mean annual temperatures and annual rainfall were compiled from He et al. (2021) for each forest and year.

All the raw data of community composition and biomass mentioned above were obtained from CERN scientific and technological resources service system (http://www.cnern.org.cn/data/initDRsearch) after online application via protocol sharing.

### 2.2 Phylogenetic tree

We constructed a phylogenetic tree for all tree species compiled. Here, we used the “mega‐tree” function in the V. PhyloMaker library (Jin & Qian, 2019) in R (R Core Team, 2015) to generate a synthetic tree. It is a phylogenetic tree generated by pruning and grafting taxa from an existing supertree (e.g., APG IV; Chase et al., 2016). The supertree we used is the most extensive dated phylogeny for vascular plants including 74, 533 species and all families of extant vascular plants (Jin & Qian, 2019); species present in our data set but missing from the “mega‐tree” were added to their respective genera using the scenario 3 approach recommended by Qian and Jin (2016).

### 2.3 Biodiversity metrics

For each quadrat and year, we calculated species richness and phylogenetic diversity. Species richness was measured as the number of species in a community, using the function “diversity” in the R vegan library (Oksanen et al., 2013). Phylogenetic diversity was measured as the sum of the lengths of total phylogenetic branches that connect species in a community, i.e., Faith's PD (Faith, 1992). These biodiversity metrics were chosen because (1) they can be directly compared in explaining the variation in biomass production since they are often highly correlated (Liu, Zhang, et al., 2015), and (2) compared with other metrics of phylogenetic diversity (e.g., mean pairwise distance MPD; Webb et al., 2002), Faith's PD might be more insensitive to unresolved nodes (i.e., polytomies) and inaccurate estimations of branch lengths (Liu et al., 2019; Mazel et al., 2016).

### 2.4 Data analysis

#### 2.4.1 Community dissimilarity and species pool size

To determine inter‐quadrat phylogenetic dissimilarity, we used an abundance‐weighted dissimilarity metric:

(1) $$W‐UniFrac=\frac{\sum_{i}^{n}b_{i}\times \left|\frac{A_{i}}{A_{T}}‐\frac{B_{i}}{B_{T}}\right|}{\sum_{j}^{n^{\prime }}d_{i}\times \left|\frac{\alpha_{j}}{A_{T}}‐\frac{B_{j}}{B_{T}}\right|},$$

where n is the number of branches in the tree, $b_{i}$ is the length of branch i, $A_{i}$ and $B_{i}$ are the numbers of individuals that descend from branch i in communities A and B, respectively, and $A_{T}$ and $B_{T}$ are the total numbers of individuals in communities A and B, respectively. $n^{\prime }$ is the number of different individuals in the two communities, $d_{j}$ is the distance from the root to individual j, while $\alpha_{j}\;$and $\beta_{j}$ are the numbers of times the sequences were observed in communities A and B, respectively (Chang et al., 2011; Lozupone et al., 2007). Using the inter‐quadrat phylogenetic dissimilarity matrix, we ran a non‐metric multidimensional scaling (NMDS) to visualize clusters. For each forest and year, we calculated the range of its inter‐quadrat phylogenetic dissimilarity (i.e., maximum phylogenetic dissimilarity minus minimum phylogenetic dissimilarity) to reflect the degree of community dissimilarity. We also used the number of total species to estimate species pool size (Karger et al., 2015).

#### 2.4.2 Generalized linear mixed‐effect models

To determine whether the strength of the biodiversity–biomass production relationship depends on forest, year, and their interaction, we constructed a series of generalized linear mixed‐effect models using the “glmer” function in the R lme4 library (Bates et al., 2015). The fixed effects included biodiversity, forest, year, and their potential interactions (e.g., biomass production ~ biodiversity + forest + year + biodiversity:forest + biodiversity:year), resulting in 18 models. The random effects included all the permanent quadrats in the three forests. The use of a gamma distribution of model residuals was validated based on the normalized scores of standardized residual deviance (*Q*‐*Q* plots). The model support was evaluated using Akaike's information criterion corrected for small sample sizes (AIC*_c_*; Burnham & Anderson, 2002; Burnham & Anderson, 2004). The model's goodness of fit was measured using marginal *R* ^2^_m_ (the variance explained by fixed effects) and conditional *R* ^2^_c_ (the variance explained by fixed and random effects) (Nakagawa et al., 2017; Nakagawa & Schielzeth, 2013). We ran the above model analysis for species richness and phylogenetic diversity, respectively (Figure S1).

We constructed two generalized linear mixed‐effect models to determine whether temperature, rainfall, species pool size, and community dissimilarity range affect biodiversity–biomass production relationships. The first model assumed an interactive effect of a factor and biodiversity on biomass production (i.e., biomass production ~ biodiversity + factor + biodiversity:factor). Correspondingly, the second model assumed their additive effect (i.e., biomass production ~ biodiversity + factor). The random effects were the same as above. Then, we calculated the information‐theoretic evidence ratio (ER) as the ratio of the model weights (i.e., interactive model vs. additive model) based on sample size‐adjusted AIC*_c_* (Saltré et al., 2016). Higher ERs (>3; Kass & Raftery, 1995) support the interactive model, meaning that the regulating effect of the factor was stronger. Furthermore, we determined the most parsimonious relationship of biomass production as a function of biodiversity, temperature, rainfall, species pool size, and community dissimilarity range. The interactive model with the highest ER was used as a base model. Because of the strong correlation between the above factors (|Spearman's ρ|>0.7; Figure S2), the more complex models were constructed with any of the variance inflation factors (VIFs) that were estimated for all parameters in a model <4 (Cade, 2015). Those models were then ranked using AIC*_c_*. Their goodness of fit was measured using the percentage of the deviance explained by the response variable (De) compared to the base model.

To determine whether phylogenetic diversity provides a better estimate than species richness to explain variation in biomass production, we compared the top‐ranked models identified for species richness and phylogenetic diversity using AIC*_c_*. All quantitative explanatory variables were standardized (i.e., mean = 0, standard deviation = 1) before model fitting. All statistical analyses were performed in R 3.5.3 (R Core Team, 2015).

## 3 RESULTS

### 3.1 Comparisons among quadrats and sites

**FIGURE 2:**
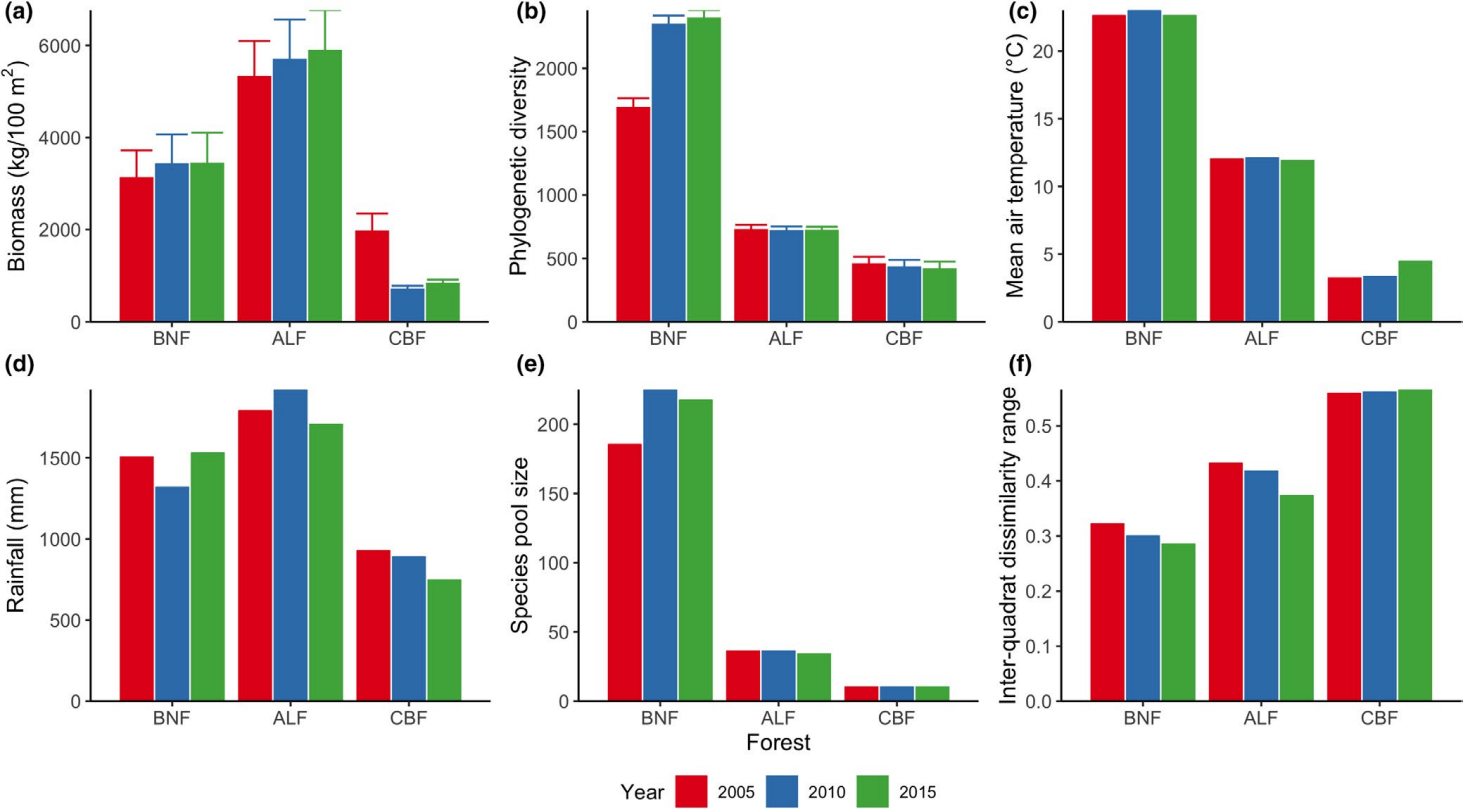
Distribution of abiotic and biotic factors in three mature natural forests in China. Biomass production (a), phylogenetic diversity (b), mean annual temperature (c), annual rainfall (d), species pool size (e), and inter‐quadrat dissimilarity range (f). The forests include Xishuangbanna tropical seasonal rainforest (BNF), Ailao Mountain subtropical evergreen broad‐leaved forest (ALF), and Changbai Mountain temperate deciduous coniferous and broad‐leaved mixed forest (CBF). Biomass production (kg/100 m^2^) was estimated using the allometric equation of the diameter at breast height (DBH) and/or tree height with the biomasses of different plant tissues (e.g., leaves, branches, stems, and roots). Phylogenetic diversity was measured using the sum of the lengths of total phylogenetic branches that connect component species in a community. Species pool size was measured using the number of species present in the permanent quadrats of each forest each year. Inter‐quadrat dissimilarity range was measured by subtracting the minimum value of phylogenetic dissimilarity between two quadrats of a forest in a year from the maximum value. Phylogenetic dissimilarity was calculated using an abundance‐weighted phylogenetic dissimilarity metric, which weights each branch length by the abundance differences of the branch along the phylogeny of the communities

Across years, variation in community composition was highest in BNF (Figure 1b), followed by ALF (Figure 1c) and CBF (Figure 1d). In terms of phylogenetic dissimilarity, community composition was distinct among the three forests (Figure 1e). Biomass production was highest in ALF and lowest in CBF and declined from 2005 to 2010 in CBF (Figure 2a). Phylogenetic diversity decreased along BNF, ALF, and CBF and increased from 2005 to 2010 in BNF (Figure 2b). The distributions of annual rainfall and mean air temperature were consistent with biomass production and phylogenetic diversity, respectively (Figure 2c,d). Moreover, species pool size also decreased with latitude (Figure 2e), while an opposite pattern occurred for the inter‐quadrat dissimilarity range, possibly due to increasing environmental heterogeneity (e.g., soil nutrients and microclimate; Figure 2f).

### 3.2 The effect of forest type and year on the biodiversity–biomass production relationship

**FIGURE 3:**
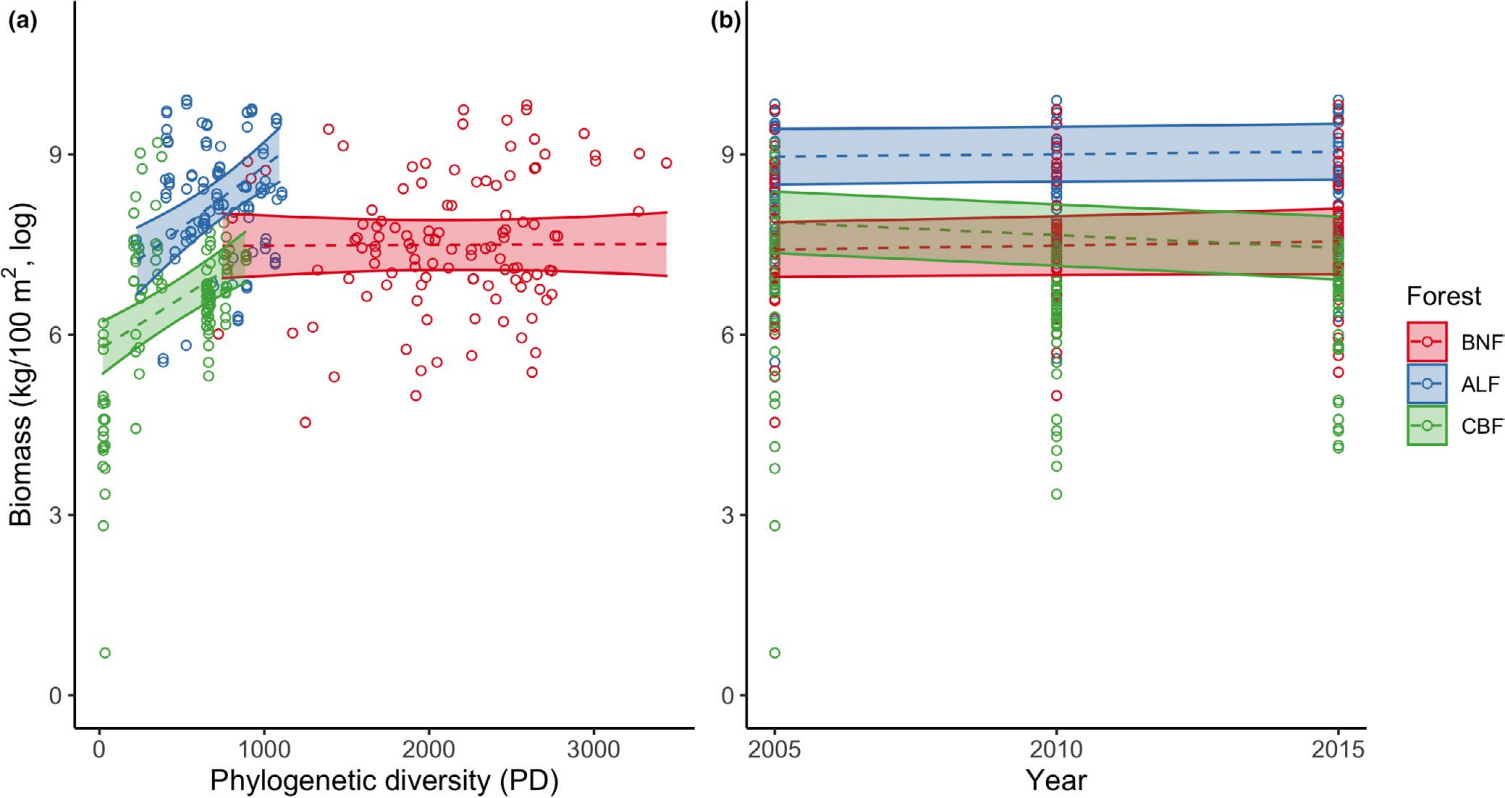
Effect plots of the generalized linear mixed‐effect model (GLMM) of biomass production as a function of the interaction between forest and phylogenetic diversity (a) and the interaction between forest and year (b). The forests include Xishuangbanna tropical seasonal rainforest (BNF), Ailao Mountain subtropical evergreen broad‐leaved forest (ALF), and Changbai Mountain temperate deciduous coniferous and broad‐leaved mixed forest (CBF). The dashed lines (95% confidence intervals shaded) represent model predictions

For the models of biomass production as a function of biodiversity, forest, year, and their potential interactions, their ranking was nearly identical between species richness and phylogenetic diversity (Table S1 and Table S2). Their top‐ranked models included biodiversity, forest, year, the interaction between biodiversity and forest, and the interaction between forest and year (*w*AIC*_c_* = 0.685 and 0.819 for species richness and phylogenetic diversity, respectively). They also accounted for comparable deviance explained in biomass production (*R* ^2^_m_ = 42.8% and 44.6% for species richness and phylogenetic diversity, respectively). However, the top‐ranked model of phylogenetic diversity was more strongly supported than that of species richness (*w*AIC*_c_* = 0.999; Table 1). As such, we only focused on phylogenetic diversity hereafter. Biomass production generally increased with phylogenetic diversity, and the relationship was stronger in ALF and CBF than in BNF (Figure 3a). Furthermore, biomass production decreased in CBF over time but increased in BNF and ALF, although trends were relatively weak (Figure 3b).

**TABLE 1:** Generalized linear mixed‐effect models (GLMMs) explain the variation in biomass production incorporating forest (F), year (Y), species richness (S), and phylogenetic diversity (PD)

Model	*k*	LL	AIC*_c_*	ΔAIC*_c_*	*w*AIC*_c_*	*R* ^2^_m_	*R* ^2^_c_
PD + F + Y + F:PD + F:Y	11	−2742.332	5507.487	0.000	0.999	44.6%	85.4%
S + F + Y + F:S + F:Y	11	−2749.557	5521.937	14.450	0.001	42.8%	85.0%

Notes

Shown are maximum log‐likelihood (LL), the estimated number of model parameters (*k*), the information‐theoretic Akaike's information criterion corrected for small samples (AIC*_c_*), the change in AIC*_c_* relative to the top‐ranked model (ΔAIC*_c_*), AIC*_c_* weighted (*w*AIC*_c_* = model probability), and the marginal and total variance explained (*R* ^2^_m_ & *R* ^2^_c_), indicating the model's goodness of fit.

### 3.3 Effects of abiotic and biotic factors on phylogenetic diversity–biomass production relationship

**FIGURE 4:**
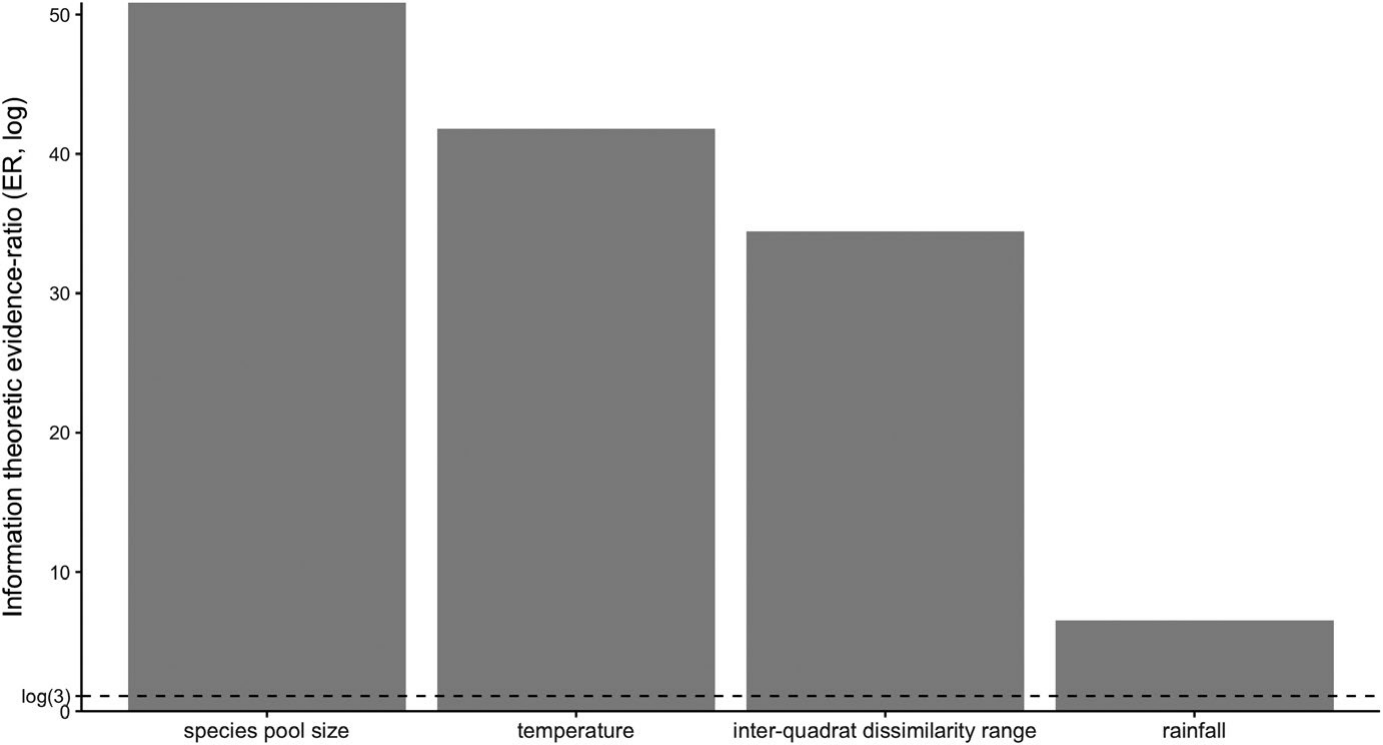
Information‐theoretic evidence ratios (ERs) comparing two generalized linear mixed‐effect models. Models are fitted on variation in the biomass production of 111 permanent quadrats (in three mature natural forests over 10 years) as a function of phylogenetic diversity and a single factor. The first model assumes an additive effect of phylogenetic diversity and the factor on biomass production (i.e., biomass ~ phylogenetic diversity + factor), whereas the second model assumes an interactive effect of phylogenetic diversity and the factor (i.e., biomass ~ phylogenetic diversity + factor + phylogenetic diversity:factor). An ER of >3 would indicate support for the interactive model. The forests include Xishuangbanna tropical seasonal rainforest (BNF), Ailao Mountain subtropical evergreen broad‐leaved forest (ALF), and Changbai Mountain temperate deciduous coniferous and broad‐leaved mixed forest (CBF)

**FIGURE 5:**
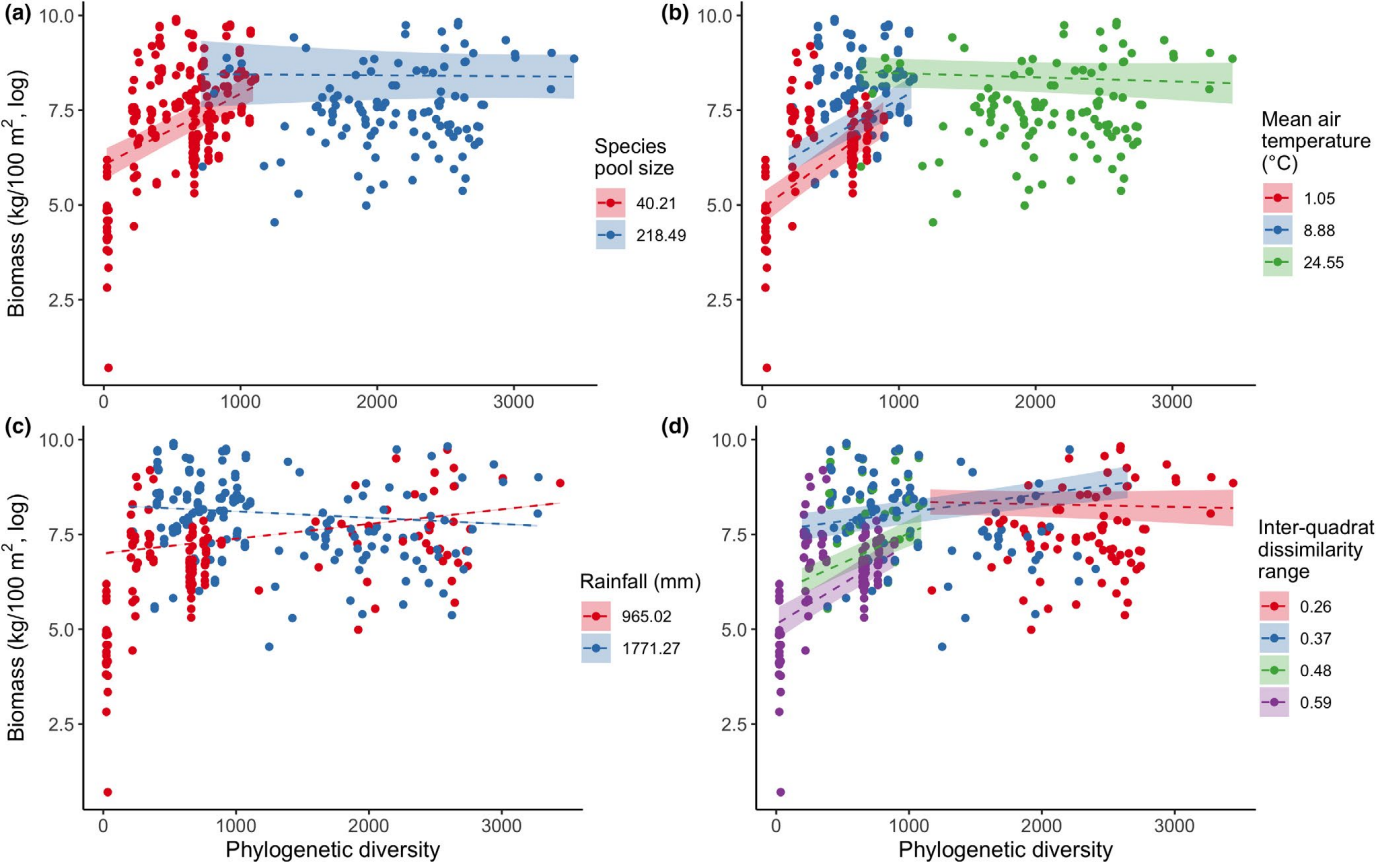
The effect of species pool size (a), mean air temperature (b), annual rainfall (c), and inter‐quadrat dissimilarity range (d) on the phylogenetic diversity–biomass production relationship. Biomass production (kg/100 m^2^) was estimated using the allometric equation of the diameter at breast height (DBH) and/or tree height with the biomasses of different plant tissues (e.g., leaves, branches, stems, and roots) for 111 permanent quadrats in three mature natural forests over 10 years. The forests include Xishuangbanna tropical seasonal rainforest (BNF), Ailao Mountain subtropical evergreen broad‐leaved forest (ALF), and Changbai Mountain temperate deciduous coniferous and broad‐leaved mixed forest (CBF). Different colors represent the different grouping of each factor with its mean value

The evidence ratio (ER) indicated that the abiotic and biotic factors considered in this study strongly influenced the phylogenetic diversity–biomass production relationship (Figure 4). The highest ER occurred for species pool size, followed by temperature, inter‐quadrat dissimilarity range, and rainfall. The relationship shifted from positive to neutral with increasing species pool size (Figure 5a), temperature (Figure 5b), and rainfall (Figure 5c), and decreasing inter‐quadrat dissimilarity range (Figure 5d). Rainfall and its interaction with phylogenetic diversity were included in the interactive model of species pool size and accounted for more than 16.3% of the deviance explained for biomass production (*w*AIC*_c_* = 0.999; Table 2).

**TABLE 2:** Generalized linear mixed‐effect models (GLMMs) explain the variation in biomass production incorporating phylogenetic diversity (PD), species pool size (P), and rainfall (R)

Model	*k*	LL	AIC*_c_*	ΔAIC*_c_*	*w*AIC*_c_*	*De*
PD + P + PD:P + R + PD:R	8	−2766.030	5548.505	0.000	0.999	16.3%
PD + P + PD:P + R	7	−2774.150	5562.644	14.139	0.001	13.7%
PD + P + PD:P	6	−2815.567	5643.392	94.887	<0.001	

Notes

Shown are maximum log‐likelihood (LL), the estimated number of model parameters (*k*), the information‐theoretic Akaike's information criterion corrected for small samples (AIC*_c_*), the change in AIC*_c_* relative to the top‐ranked model (ΔAIC*_c_*), AIC*_c_* weighted (*w*AIC*_c_* = model probability), and the percentage of deviance additionally explained (*De*) compared to the base model (i.e., PD + P + PD:P), which serves as a measure of the model's goodness of fit.

## 4 DISCUSSION

The biodiversity–biomass production relationship strengthens with environmental stress (Ratcliffe et al., 2017) and time (Tatsumi, 2020), and these regulating effects might be mediated by both abiotic and biotic factors (Liu et al., 2021). Our results reveal that species pool size and rainfall account for phylogenetic diversity–biomass production relationships found in natural forests of China.

### 4.1 Strong phylogenetic diversity‐biomass production relationship

We found a positive relationship between phylogenetic diversity and biomass production after accounting for forest type and year, a finding that is in line with previous studies (Lasky et al., 2014; Satdichanh et al., 2019). Moreover, the top‐ranked biomass production model included the interaction between phylogenetic diversity and forest type, suggesting phylogenetic diversity–biomass production relationships might be context dependent; the relationship we found was much stronger in the subtropical and temperate forests than in tropical forests. Similar trends have been shown for the effects of functional diversity on productivity, where the effect is higher in boreal forests than in temperate forests (e.g., Paquette & Messier, 2011). Our results generally agree with our expectation that the relationship between phylogenetic diversity and biomass production will strengthen with increasing environmental stress (Liu et al., 2021; Mulder et al., 2001).

### 4.2 No evidence for the time dependency of biodiversity–biomass production relationship

We found the interaction between phylogenetic diversity and year did not remain in our top‐ranked models, which was inconsistent with our expectation that the effect of biodiversity on biomass production would increase with time (Cardinale et al., 2007). To our knowledge, there is only one study that investigates how phylogenetic diversity–biomass production relationships may change with time in forest ecosystems (i.e., Satdichanh et al., 2019). Surprisingly, they found a stronger relationship in younger trees. However, they evaluated the relationship at sites along a chronosequence of succession (i.e., substituting space for time), which requires accounting for other confounding effects, such as community composition and abiotic factors (Isbell et al., 2018). In our study, the sampling regime along the temporal scale might be one reason for the lack of temporal effects (i.e., our dataset only included three sampling points over 10 years). Indeed, in our compiled dataset, BNF was surveyed from 2004 to 2010, in addition to 2015, and in more permanent quadrats (99 in the dataset). Therefore, we compared the additive and interactive models in BNF and found strong support for the interactive model (*w*AIC*_c_* = 0.966; Table S3). In general, phylogenetic diversity increased biomass production with time (Figure S3), although it was estimated using mean pairwise distance (MPD, i.e., the average phylogenetic distance separating all pairs of species on a phylogenetic tree; Webb et al., 2002). More research is needed to generalize the effect of biodiversity on the biomass production of forest ecosystems across time.

### 4.3 Abiotic and biotic factors regulate biodiversity–biomass production relationship

Our results found that temperature and rainfall underpin a context‐dependent phylogenetic diversity–biomass production relationship, generally agreeing with previous studies (Ammer, 2019; Fei et al., 2018; Hisano & Chen, 2020; Jactel et al., 2018; Wang & Ali, 2021). When considered separately, however, the effect was stronger for temperature than rainfall, which is in contrast with previous studies that imply water availability as a more important driver of context‐dependent biodiversity effects (e.g., Fei et al., 2018; Jactel et al., 2018; Hisano & Chen, 2020; although see Wang & Ali, 2021). Our study is also novel because biotic factors, such as species pool size and community dissimilarity range in forest ecosystems, had a strong effect on the biodiversity–biomass production relationship, although species pool size showed the strongest influence when considered alone. Armitage (2016) reports that species pool might account for the varying relationship between biodiversity and ecosystem functioning for bacterial isolates across a natural successional gradient. Our results partly agree with this finding because we found evidence of species pool size influencing the phylogenetic diversity–biomass production relationship across time in BNF (Table S4). Interestingly, increasing species pool size in temperate forests strengthened the relationship between phylogenetic diversity and biomass production with time (Figure S4), but the relationship was weakened in the tropical forest.

Moreover, we found that species pool size, rainfall, and their interactions with phylogenetic diversity constituted the top‐ranked model. This, in turn, supports the importance of water availability on the biodiversity–biomass production relationship in natural forest ecosystems. However, the top‐ranked model was less supported when compared with the interactive model of forest (i.e., biomass ~ PD + Forest + PD:Forest; *w*AIC*_c_* < 0.001; Table S5). This result implies that some critical factors, such as soil and leaf microorganisms, are missing in our study (Laforest‐Lapointe et al., 2017; Liang et al., 2019). Collectively, however, our results emphasize that both abiotic and biotic factors are required to understand variation in the biodiversity–biomass production relationship at our study sites (Liu et al., 2021).

### 4.4 Strong phylogenetic diversity–biomass relationship in different forests and years

Phylogenetic diversity was a stronger predictor of biomass production compared to species richness. This finding corroborates previous empirical evidence (see Cadotte et al., 2009; Cardinale et al., 2015; Genung et al., 2014; Hao et al., 2018; Liang et al., 2019; Liu et al., 2018; Liu, Zhang, et al., 2015). One reason for this trend might be that it has close connections with functional traits important for biomass production. For example, hydraulics‐related functional traits are important for productivity in a forest biodiversity experiment (Bongers et al., 2021), where such traits typically have strong phylogenetic signals (Liu, Xu, et al., 2015). We, therefore, recommend future studies use phylogenetic diversity metrics instead of species richness to assess biodiversity–biomass production relationships, especially when functional traits are not available.

## 5 CONCLUSIONS

Our study contributes to understanding the varying relationships between biodiversity and biomass production often observed in natural ecosystems. Our results support a strong context‐dependent phylogenetic diversity–biomass production relationship in natural forest ecosystems. In general, the relationship between phylogenetic diversity and biomass production strengthened with environmental stress. More importantly, our results suggest abiotic and biotic factors, especially rainfall and species pool size, underlie the relationship; increasing species pool size and rainfall was associated with the decreasing effect of phylogenetic diversity on biomass production. Moreover, the biodiversity metrics that incorporate phylogenetic relationships between species or functional traits should be given priority when considering the biodiversity–ecosystem functioning relationship in natural forests.

Nevertheless, the small number of sampling units spanning our environmental stress gradient and temporal scales is one caveat to consider. Our sampling regime might have underestimated the mediating effects of species pool size and rainfall on the relationship between biodiversity and biomass production in natural forest ecosystems. More studies are required to evaluate further the biodiversity–biomass production relationship across more extensive gradients of species pool size and rainfall.

## CONFLICTS OF INTEREST

None declared.

## AUTHOR CONTRIBUTIONS

**Jia‐Jia Liu:** Conceptualization (lead); Data curation (lead); Formal analysis (lead); Funding acquisition (equal); Investigation (lead); Methodology (lead); Project administration (lead); Resources (lead); Software (lead); Supervision (equal); Validation (equal); Visualization (lead); Writing – original draft (lead); Writing – review & editing (equal). **Kevin S. Burgess:** Conceptualization (supporting); Data curation (supporting); Formal analysis (supporting); Funding acquisition (supporting); Investigation (supporting); Methodology (supporting); Project administration (supporting); Resources (supporting); Software (supporting); Supervision (lead); Validation (equal); Visualization (supporting); Writing – original draft (supporting); Writing – review & editing (lead). **Xue‐Jun Ge:** Conceptualization (supporting); Data curation (supporting); Formal analysis (supporting); Funding acquisition (equal); Investigation (supporting); Methodology (supporting); Project administration (lead); Resources (supporting); Software (supporting); Supervision (lead); Validation (supporting); Visualization (supporting); Writing – original draft (supporting); Writing – review & editing (equal).

## Supporting information

Supplementary Material

## Data Availability

The essential data and R script for reproducing the data analyses of this study are deposited in http://www.scidb.cn/doi/10.11922/sciencedb.01652. https://doi.org/10.11922/sciencedb.01652.
