# Supplementary material for: Species pool size and rainfall account for the relationship between biodiversity and biomass production in natural forests of China
Source: Ecol Evol. 2022 Apr 21;12(4):e8838. doi: 10.1002/ece3.8838 (PMC9022444; doi:10.1002/ece3.8838)

**Table S1.** **Generalized linear mixed-effects models (GLMMs) explain the variation in biomass production incorporating forest (F), year (Y), and phylogenetic diversity (PD).**

| **Model** | ***k*** | **LL** | **AIC*_c_*** | **ΔAIC*_c_*** | ***w*AIC*_c_*** | **R^2^_m_** | **R^2^_c_** |
| --- | --- | --- | --- | --- | --- | --- | --- |
| F + PD + Y + F:PD + F:Y | 11 | -2742.332 | 5507.487 | 0.000 | 0.819 | 44.6% | 85.4% |
| F + PD + Y + F:PD + F:Y + PD:Y | 12 | -2743.266 | 5511.508 | 4.020 | 0.110 | 44.3% | 84.9% |
| F + PD + Y + F:PD + F:Y + PD:Y + F:PD:Y | 14 | -2741.552 | 5512.425 | 4.938 | 0.069 | 44.3% | 85.4% |
| F + PD + Y + F:PD + PD:Y | 10 | -2749.503 | 5519.688 | 12.201 | 0.002 | 43.6% | 85.0% |
| F + PD + Y + F:PD | 9 | -2752.677 | 5523.911 | 16.424 | <0.001 | 42.9% | 84.8% |
| F + PD + F:PD | 8 | -2754.549 | 5525.543 | 18.056 | <0.001 | 42.1% | 84.6% |
| F + PD + Y + F:Y | 9 | -2768.098 | 5554.752 | 47.265 | <0.001 | 39.1% | 82.8% |
| F + PD + Y + F:Y + PD:Y | 10 | -2767.644 | 5555.971 | 48.484 | <0.001 | 39.6% | 82.9% |
| F + PD + Y | 7 | -2775.695 | 5565.734 | 58.247 | <0.001 | 37.4% | 82.4% |
| F + PD + Y + PD:Y | 8 | -2775.219 | 5566.882 | 59.395 | <0.001 | 37.7% | 82.4% |
| F + Y + F:Y | 8 | -2776.425 | 5569.295 | 61.808 | <0.001 | 37.2% | 82.4% |
| F + PD | 6 | -2781.086 | 5574.430 | 66.943 | <0.001 | 35.4% | 81.9% |
| F + Y | 6 | -2787.819 | 5587.897 | 80.409 | <0.001 | 34.6% | 81.7% |
| F | 5 | -2789.042 | 5588.268 | 80.780 | <0.001 | 33.9% | 81.6% |
| PD + Y | 5 | -2864.631 | 5739.445 | 231.958 | <0.001 | 15.3% | 77.1% |
| PD + Y + PD:Y | 6 | -2864.633 | 5741.523 | 234.036 | <0.001 | 15.3% | 77.2% |
| PD | 4 | -2867.475 | 5743.072 | 235.585 | <0.001 | 11.8% | 75.9% |
| Y | 4 | -2878.373 | 5764.869 | 257.381 | <0.001 | 0.0% | 72.2% |

*Notes:* Shown are maximum log-likelihood (LL), the estimated number of model parameters (*k*), the information-theoretic Akaike’s information criterion corrected for small samples (AIC*_c_*), the change in AIC*_c_* relative to the top-ranked model (ΔAIC*_c_*), AIC*_c_* weighted (*w*AIC*_c_* = model probability), and the marginal and total variance explained (*R^2^_m_* & *R^2^_c_*) as the measure of the model’s goodness-of-fit.

**Table S2.** **Generalized linear mixed-effects models (GLMMs) explain the variation in biomass production incorporating forest (F), year (Y), and species richness (*S*).**

| **Model** | ***k*** | **LL** | **AIC*_c_*** | **ΔAIC*_c_*** | ***w*AIC*_c_*** | **R^2^_m_** | **R^2^_c_** |
| --- | --- | --- | --- | --- | --- | --- | --- |
| F + *S* + Y + F:*S* + F:Y | 11 | -2749.557 | 5521.937 | 0.000 | 0.685 | 42.8% | 85.0% |
| F + *S* + Y + F:*S* + F:Y + *S*:Y | 12 | -2749.554 | 5524.083 | 2.146 | 0.234 | 42.8% | 85.0% |
| F + *S* + Y + F:*S* + F:Y + *S*:Y + F:*S*:Y | 14 | -2748.612 | 5526.545 | 4.608 | 0.068 | 42.6% | 85.0% |
| F + *S* + Y + F:*S* + *S*:Y | 10 | -2754.854 | 5530.390 | 8.453 | 0.010 | 41.9% | 84.8% |
| F + *S* + Y + F:*S* | 9 | -2757.871 | 5534.299 | 12.362 | 0.001 | 41.4% | 84.6% |
| F + *S* + F:*S* | 8 | -2760.177 | 5536.799 | 14.862 | <0.001 | 40.5% | 84.5% |
| F + *S* + Y + F:Y | 9 | -2774.267 | 5567.092 | 45.155 | <0.001 | 37.8% | 82.4% |
| F + *S* + Y + F:Y + *S*:Y | 10 | -2774.107 | 5568.898 | 46.961 | <0.001 | 37.9% | 82.5% |
| F + Y + F:Y | 8 | -2776.425 | 5569.295 | 47.358 | <0.001 | 37.2% | 82.4% |
| F + *S* + Y + *S*:Y | 8 | -2780.509 | 5577.462 | 55.525 | <0.001 | 36.5% | 82.1% |
| F + *S* + Y | 7 | -2782.009 | 5578.362 | 56.425 | <0.001 | 36.1% | 82.0% |
| F + *S* | 6 | -2786.126 | 5584.510 | 62.573 | <0.001 | 34.4% | 81.6% |
| F + Y | 6 | -2787.819 | 5587.897 | 65.960 | <0.001 | 34.6% | 81.7% |
| F | 5 | -2789.042 | 5588.268 | 66.331 | <0.001 | 33.9% | 81.6% |
| *S* + Y | 5 | -2865.478 | 5741.139 | 219.202 | <0.001 | 13.9% | 76.6% |
| *S* + Y + *S*:Y | 6 | -2865.474 | 5743.205 | 221.268 | <0.001 | 13.7% | 76.6% |
| *S* | 4 | -2868.872 | 5745.865 | 223.928 | <0.001 | 9.7% | 75.2% |
| Y | 4 | -2878.373 | 5764.869 | 242.932 | <0.001 | 0.0% | 72.2% |

*Notes:* Shown are maximum log-likelihood (LL), the estimated number of model parameters (*k*), the information-theoretic Akaike’s information criterion corrected for small samples (AIC*_c_*), the change in AIC*_c_* relative to the top-ranked model (ΔAIC*_c_*), AIC*_c_* weighted (*w*AIC*_c_* = model probability), and the marginal and total variance explained (*R^2^_m_* & *R^2^_c_*) as the measure of the model’s goodness-of-fit.

**Table S3.** **Generalized linear mixed-effects models (GLMMs) explain the variation in biomass production incorporating phylogenetic diversity (MPD) and year (Y).**

| **Model** | ***k*** | **LL** | **AIC*_c_*** | **ΔAIC*_c_*** | ***w*AIC*_c_*** | **R^2^_m_** | **R^2^_c_** |
| --- | --- | --- | --- | --- | --- | --- | --- |
| MPD + Y + MPD:Y | 6 | -5228.151 | 10468.428 | 0.000 | 0.966 | 5.1% | 74.0% |
| MPD + Y | 5 | -5232.511 | 10475.112 | 6.685 | 0.034 | 2.8% | 73.4% |

*Notes:* Phylogenetic diversity is estimated using mean pairwise distance (MPD), which averages the distance separating all pairs of species in the phylogenetic tree. Shown are maximum log-likelihood (LL), the estimated number of model parameters (*k*), the information-theoretic Akaike’s information criterion corrected for small samples (AIC*_c_*), the change in AIC*_c_* relative to the top-ranked model (ΔAIC*_c_*), AIC*_c_* weighted (*w*AIC*_c_* = model probability), and the marginal and total variance explained (*R^2^_m_* & *R^2^_c_*) as the measure of the model’s goodness-of-fit.

**Table S4.** **Generalized linear mixed-effects models (GLMMs) explain the variation in biomass production incorporating phylogenetic diversity (MPD) and species pool size (P).**

| **Model** | ***k*** | **LL** | **AIC*_c_*** | **ΔAIC*_c_*** | ***w*AIC*_c_*** | **R^2^_m_** | **R^2^_c_** |
| --- | --- | --- | --- | --- | --- | --- | --- |
| MPD + P + MPD:P | 6 | -5224.339 | 10460.805 | 0.000 | 0.759 | 3.5% | 73.7% |
| MPD + P | 5 | -5226.504 | 10463.098 | 2.293 | 0.241 | 2.3% | 73.4% |

*Notes:* Shown are maximum log-likelihood (LL), the estimated number of model parameters (*k*), the information-theoretic Akaike’s information criterion corrected for small samples (AIC*_c_*), the change in AIC*_c_* relative to the top-ranked model (ΔAIC*_c_*), AIC*_c_* weighted (*w*AIC*_c_* = model probability), and the marginal and total variance explained (*R^2^_m_* & *R^2^_c_*) as the measure of the model’s goodness-of-fit.

**Table S5. Generalized linear mixed-effects models (GLMMs) explain the variation in biomass production incorporating phylogenetic diversity (PD), forest (F), species pool size (P), and rainfall (R).**

| **Model** | ***k*** | **LL** | **AIC*_c_*** | **ΔAIC*_c_*** | ***w*AIC*_c_*** | ***De*** |
| --- | --- | --- | --- | --- | --- | --- |
| PD + F + PD:F | 8 | -2754.549 | 5525.543 | 0.000 | 0.999 | 7.9% |
| PD + P + PD:P +  R + PD:R | 8 | -2766.030 | 5548.505 | 22.962 | <0.001 | 0.0% |

*Notes:* Shown are maximum log-likelihood (LL), the estimated number of model parameters (*k*), the information-theoretic Akaike’s information criterion corrected for small samples (AIC*_c_*), the change in AIC*_c_* relative to the top-ranked model (ΔAIC*_c_*), AIC*_c_* weighted (*w*AIC*_c_* = model probability), and the percentage of deviance additional explained (*De*) compared to the base model (i.e., PD + P + PD:P + R + PD:R), which serves as a measure of the model’s goodness-of-fit.

**Figure S1. Relationship between species richness and phylogenetic diversity in three mature natural forests in China.**


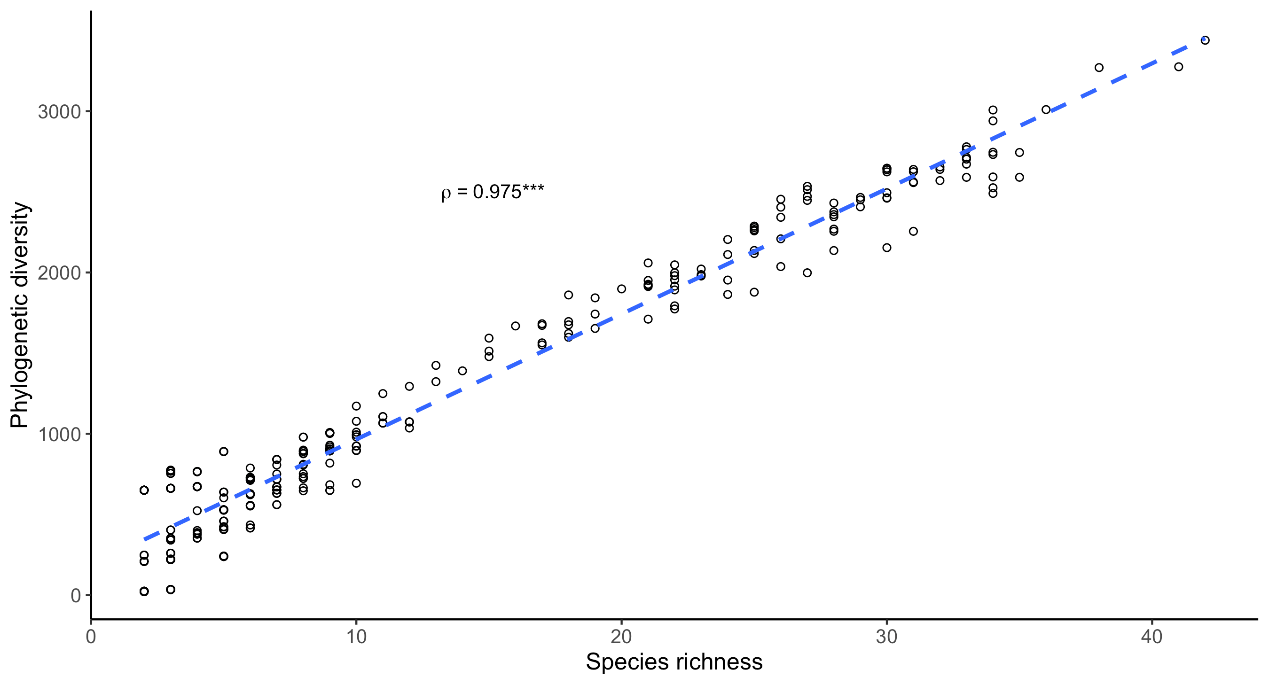


The forests include Xishuangbanna tropical seasonal rainforest (BNF), Ailao Mountain subtropical evergreen broad-leaved forest (ALF), and the Changbai Mountain temperate deciduous coniferous and broad-leaved mixed forest (CBF). Solid line is their linear regression line. Shown in text is the correlation coefficient (Spearman’s $\rho$) and its statistical significance (* *p* < 0.05, ** *p* < 0.01, *** *p* < 0.001).

**Figure S2. Correlation matrix of abiotic and biotic factors in three mature natural forests in China.** The forests include Xishuangbanna tropical seasonal rainforest (BNF), Ailao Mountain subtropical evergreen broad-leaved forest (ALF), and the Changbai Mountain temperate deciduous coniferous and broad-leaved mixed forest (CBF). Factors include: mean annual temperature (°C), annual rainfall (mm), species pool size, and inter-quadrat dissimilarity range. Correlation coefficients, and associated statistical significance (* *p* < 0.05, ** *p* < 0.01, *** *p* < 0.001), are shown in text.


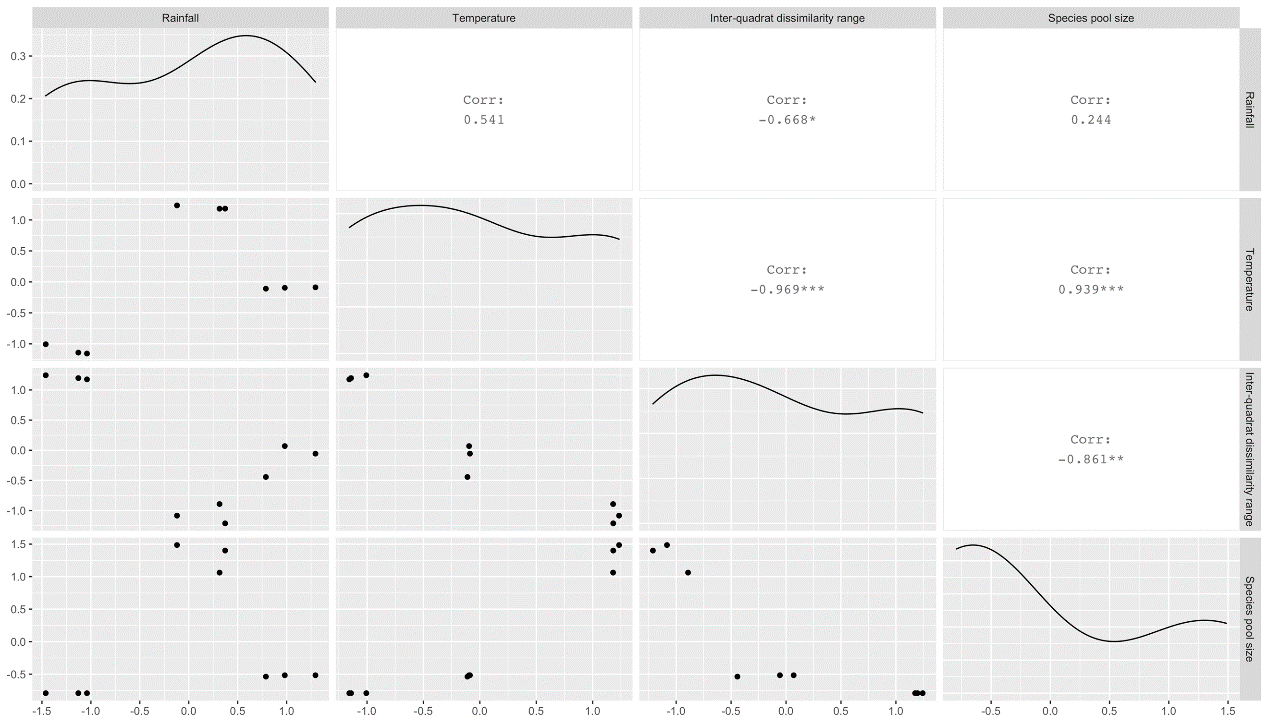


**Figure S3. Relationship between phylogenetic diversity and biomass production in Xishuangbanna tropical seasonal rainforest (BNF).** Biomass production (kg/100 m^2^) was estimated using the allometric equation of the diameter at breast height (DBH) and/or tree height with the biomasses of different plant tissues (e.g., leaves, branches, stems, and roots) for 99 permanent quadrats in BNF across time. Phylogenetic diversity was measured using the mean phylogenetic distance separating all pairs of component species in a community (MPD). The dashed lines represent the linear regression (95% confidence intervals shaded).


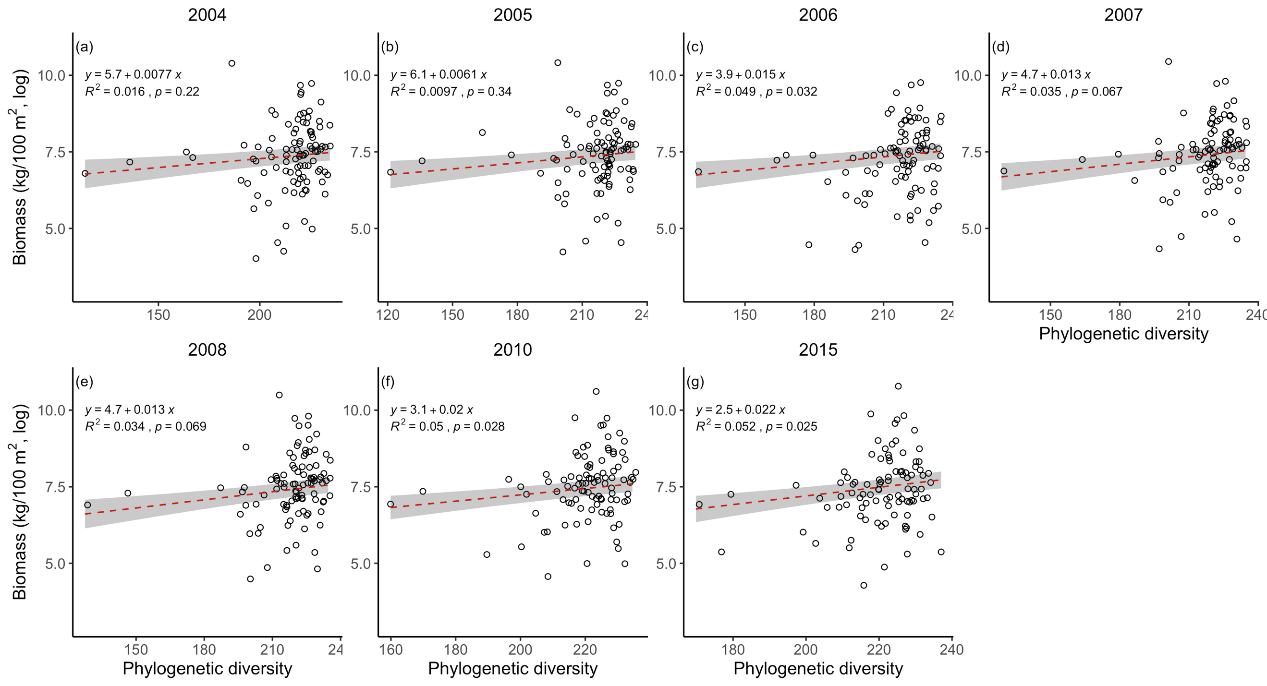


**Figure S4.** **Regulating effects of species pool size on the phylogenetic diversity-biomass production relationship in Xishuangbanna tropical seasonal rainforest (BNF).** Biomass production (kg/100 m^2^) was estimated using the allometric equation of the diameter at breast height (DBH) and/or tree height with the biomasses of different plant tissues (e.g., leaves, branches, stems, and roots) for 99 permanent quadrats in BNF across time (see above). Phylogenetic diversity was measured using the mean phylogenetic distance separating all pairs of component species in a community (MPD). Species pool size was measured using the number of species present in the permanent quadrats each year. Different colors represent the different grouping of species pool size with their mean value.


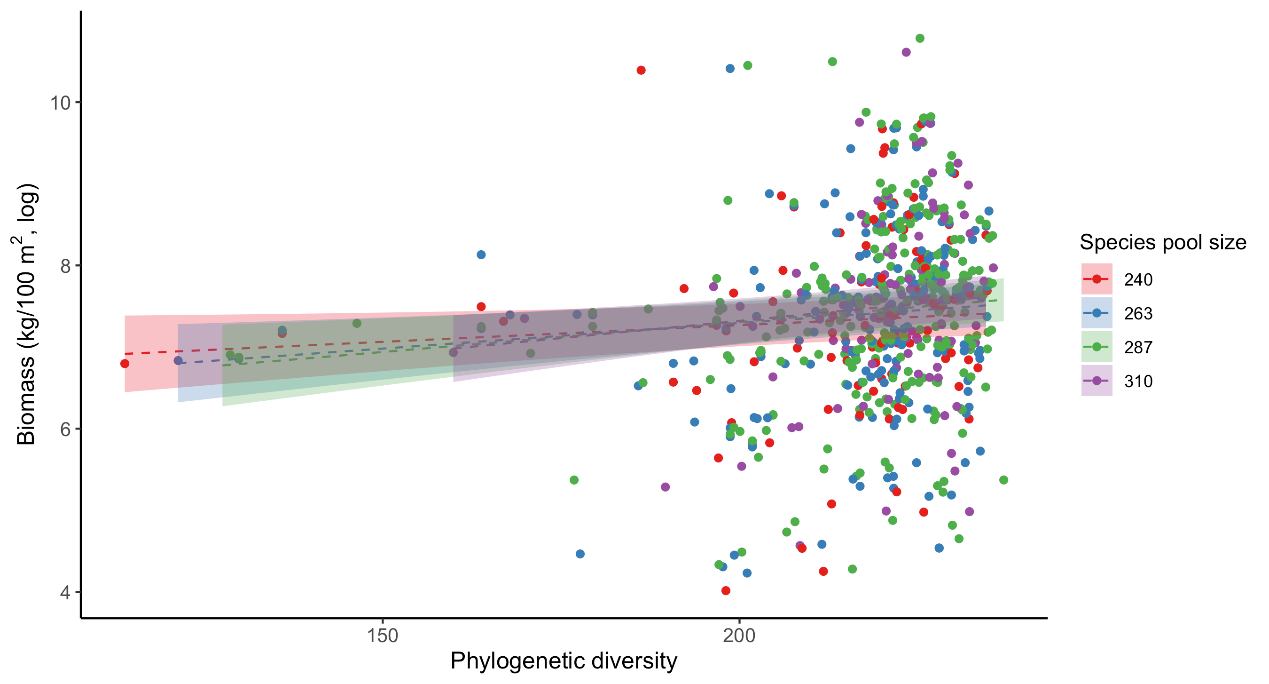

Supplement: Supplementary file 1 — Supplementary Material [file ECE3-12-e8838-s001.docx]
